# Unlocking plant growth-promoting traits of endophytic actinobacteria isolated from *Anacyclus pyrethrum*, an endemic medicinal plant of the Aguelmam azegza region, Morocco

**DOI:** 10.3389/fmicb.2025.1682456

**Published:** 2025-09-30

**Authors:** Rachid Aguennouz, Yassine Aallam, Abdelmajid Haddioui, Hanane Hamdali

**Affiliations:** 1 Laboratory of Agro-Industrial and Medical Biotechnology, Faculty of Sciences and Technology, University of Sultan Moulay Slimane, Beni-Mellal, Morocco; 2 Agro-Biosciences Program, College of Sustainable Agriculture and Environmental Sciences, Mohammed VI Polytechnic University, Ben Guerir, Morocco

**Keywords:** pyrethrum, endophytic actinobacteria, plant growth promoting (PGP), phosphate solubilization, Indole-3-Acetic Acid (IAA), antimicrobial activity

## Abstract

**Introduction:**

The present study reports, for the first time to our knowledge, the isolation and characterization of endophytic actinobacteria from the medicinal plant *Anacyclus pyrethrum*.

**Methods:**

A total of 100 endophytic actinobacterial strains were isolated from root tissues. Based on their distinct morphological characteristics observed on ISP2 and Bennett media, thirteen representative isolates were selected for further screening of their plant growth-promoting traits and antimicrobial activities. These isolates were evaluated for their ability to solubilize inorganic phosphate, produce Indole-3-Acetic Acid (IAA), and exhibit antibacterial and antifungal activities. Antibacterial assays targeted *Escherichia coli*, *Klebsiella pneumoniae*, and *Staphylococcus aureus*, while antifungal activity was assessed against *Fusarium fujikuroi*.

**Results:**

Approximately 80% of the selected isolates displayed plant growth-promoting potential under in vitro conditions. Five isolates (38.46%) tested positive for IAA production, with *AGS05* and *AGS08* producing the highest concentrations (87.54 μg/mL and 89.79 μg/mL, respectively). Moderate IAA production was observed in *AGS13* (59.23 μg/mL) and *AGS09* (35.31 μg/mL). Furthermore, nine isolates (69.23%) exhibited phosphate-solubilizing abilities, as evidenced by clear halo formations on Synthetic Minimum Medium (SMM) containing 0.5 g/L of tricalcium phosphate (TCP) or rock phosphate (RP) as the sole phosphorus sources. Quantitative assessment of phosphate solubilization in liquid SMM revealed that phosphate release increased significantly by day 7 across all tested strains. *AGS08* released the highest amount of soluble phosphate from TCP (47.6 μg/mL, pH 3.55), followed by *AGS03* (41.2 μg/mL, pH 3.85) and *AGS13* (39.6 μg/mL, pH 3.75). Regarding RP solubilization, *AGS10* exhibited the highest P release (7.8 μg/mL, pH 3.4), followed by *AGS09* (7.0 μg/mL, pH 3.9) and AGS08 (6.4 μg/mL, pH 4.5). A consistent pH reduction was observed across all treatments, indicating microbial acidification during phosphate solubilization. Based on 16S rRNA gene sequencing and phylogenetic analysis, three isolates with multiple PGP traits were identified as *Streptomyces* species: *S. albogriseolus* [*AGS8 (By8)*] and *S.* var*iabilis* [*AGS5 (By5a)* and *AGS10 (By10)*].

**Discussion:**

These findings demonstrate that Anacyclus pyrethrum is a valuable reservoir of endophytic actinobacteria with remarkable plant growth-promoting and antimicrobial properties. The selected strains represent promising candidates for development as bioinoculants and biocontrol agents in sustainable agricultural systems.

## Introduction

Understanding and managing plant microbe interactions can offer substantial benefits for improving crop productivity, particularly under stress conditions. The presence of microbial cells within plant tissues was first described by de Bary (1866), who coined the term “endophytic.”

These microorganisms, which colonize plant tissues inter and/or intracellularly without causing harm (Schulz and Boyle, 2006), are now recognized as ubiquitous in the plant kingdom, with representatives from all three domains of life Bacteria, Archaea and Eukarya forming endophytic associations under various climatic conditions (Hirsch and Mauchline, 2012).

Endophytic actinobacteria are among the most prominent microbial groups associated with plants. They have been detected in nearly all plant species studied, where they contribute significantly to enhanced nutrient use efficiency and increased tolerance to biotic and abiotic stresses (Passari et al., 2015). These microbes can complete at least part of their life cycle within host tissues, and their diversity is influenced by plant species, stress levels, and soil characteristics (Nalini and Prakash, 2017; Nguyen et al., 2025).

Actinobacteria, commonly referred to as actinomycetes, are Gram-positive bacteria with high G+C content, currently comprising 5 subclasses, 6 orders and 14 suborders (Barka et al., 2016). Recent taxonomic updates by Salam et al. (2020) further expanded the group to 6 classes, 46 orders and 79 families, including 16 new orders and 10 new families. The most frequently isolated endophytic actinobacteria belong to the genus *Streptomyces* (Barka et al., 2016), along with other genera such as *Actinomadura*, *Actinoplanes*, *Micromonospora*, *Frankia*, *Nocardia*, *Saccharopolyspora* and *Verrucosispora*, all known for their ecological and biotechnological importance (Martínez-Hidalgo et al., 2014).

Ethnobotanical plants, in particular, have been identified as valuable sources of potent endophytic microorganisms, and it has been shown that the diversity of actinobacteria can vary across different plant tissues (Jangra et al., 2024).

Several studies have reviewed the diversity of endophytic actinobacteria in medicinal plants (Nguyen et al., 2025; Fasusi et al., 2021), and investigations by Du et al. (2013) reported the isolation of 600 actinobacteria from 37 medicinal plants, representing 34 genera and several unknown taxa. However, not all plants harbor endophytic actinomycetes (Strobel et al., 2004). Dinesh et al. (2017) observed that actinobacteria could colonize various plant organs and that colonization patterns depend on plant-microbe interactions.

Endophytic actinobacteria can promote plant growth directly, through the production of phytohormones and solubilization of minerals like phosphorus (Singh et al., 2023), or indirectly, by protecting plants against phytopathogens via antimicrobial production and induction of host resistance (Passari et al., 2017).

Given their increasing relevance, endophytic actinobacteria are being explored as biofertilizers and biocontrol agents (Jangra et al., 2024). Endophyte-based biofertilizers can improve rhizosphere conditions and enhance nutrient availability, particularly for nitrogen, phosphorus and potassium (Xie et al., 2023). Phosphorus, although essential for plant processes such as glucose transport, cell proliferation and organ development (Hamdali et al., 2008a), is poorly available in soils due to fixation and low solubility (Cordell et al., 2009). While chemical fertilizers are widely used, they account for 80–90% of global phosphate consumption in agriculture (Cordell et al., 2009). Endophytic actinobacteria can increase phosphorus availability through acidification, chelation, redox reactions and organic phosphorus mineralization (Cordell et al., 2009).

Hamdali et al. (2008a) also demonstrated that phosphate solubilizing actinobacteria enhanced wheat growth, and several species have shown the ability to solubilize forms such as tricalcium phosphate and rock phosphate (Cordell et al., 2009). Despite growing interest, few studies have investigated the beneficial roles of endophytic actinobacteria from plants, particularly in the context of biofertilizer development (Barka et al., 2016). These microbial inoculants represent a promising alternative to conventional agriculture, especially in developing countries in Asia and Africa, where sustainable agriculture is critical (Singh et al., 2023; Batista and Singh, 2021).

The present study is the first to report the isolation and characterization of endophytic actinobacteria from *Anacyclus pyrethrum*, a medicinal plant endemic to North Africa (Fennane et al., 2014). The leaves are finely dissected, delicate, and pubescent, contributing to its distinctive morphology. The inflorescences bear yellow-centered capitula composed of white ray florets on the inner side and purplish hues on the outer margins (Fennane et al., 2014). Its fruits (achenes) are glabrous or may exhibit a faint crown structure (Cazin, 1850). The flowering period extends from May to August (Humphries, 1979).

This species appears to be a rich reservoir for actinobacteria, many of which display promising traits as plant growth-promoting microorganisms and potential biocontrol agents. These findings highlight the potential use of endophytic actinobacteria from *A. pyrethrum* as bioinoculants to support sustainable agricultural practices.

## Materials and methods

### Collection of *Anacyclus pyrethrum*—root samples

**Figure 1:**
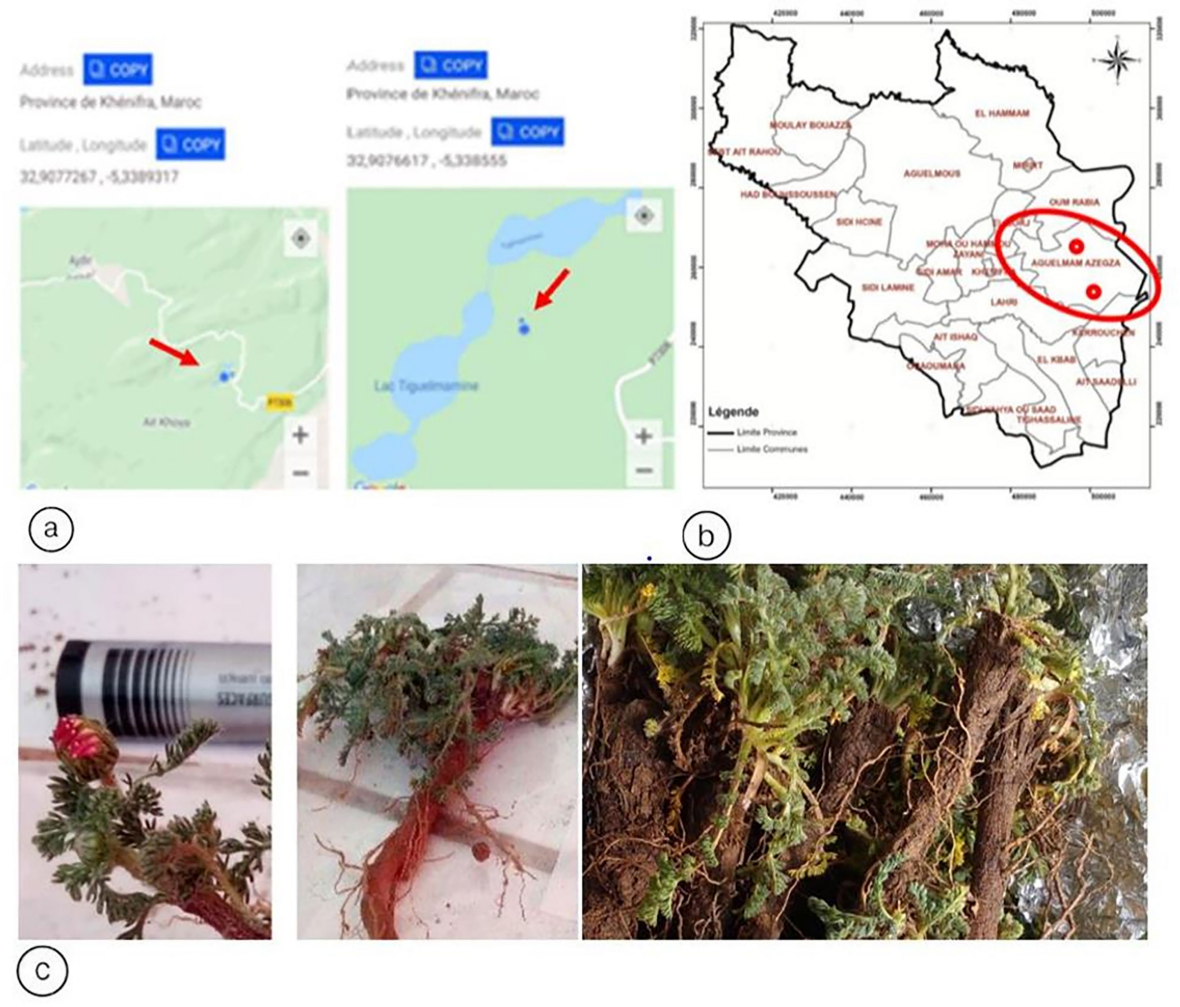
**(a)** Administrative division of the Beni Mellal-Khenifra region (Morocco), with a focus on Khenifra Province. **(b)** Biogeographic location of the sampling sites 1 & 2. **(c)** Morphological appearance: Flowers, ray florets and root of *Anacyclus pyrethrum* var.*pyrethrum* “*Igendas*” roots.

The samples were collected from two different sites located in the Rural Commune of Aguelmam Azegza in the Central Middle Atlas of Morocco, within the biogeographic zone of Ait Boumzil (Site 1) (Latitude: 32.9076617°N; Longitude: −5.338555°W), and in the biogeographic zone of Ajdir des Izayanes (Site 2) (Latitude: 32.9277267°N; Longitude: −5.3389317°W). Plant samples were collected, placed in sterile plastic bags, transported to the laboratory, and subjected to isolation procedures. All samples were collected aseptically using the following steps: using a sterile spade, we dug at the plant tip of the study area to a depth of 20 cm (Figure 1).

### Isolation of endophytic actinobacteria

These explants are washed in running tap water to remove adhered epiphytes, soil debris or dust particles on the surface, followed by surface sterilization using one or more different surface sterilizing agents. The root samples were air dried for 24 h at room temperature and then washed in running tap water to remove adhered epiphytes, soil debris or dust particles on the surface, followed by surface sterilization using one or more different surface sterilizing agents. After drying, the samples were subjected to a five-step surface sterilization procedure (Schulz et al., 1993; Gao et al., 2022): a 4 min–10 min wash in 5% NaOCl (disinfectant), followed by a 10-min wash in 22.5% sodium thiosulfate (Na_2_S_2_O_3_), a 5 min wash in 75% ethanol, a wash in sterile water, and a final rinse in 10% sodium bicarbonate (NaHCO_3_) for 10 min. After being thoroughly dried under sterile conditions, the surface sterilized tissues were subjected to continuous drying at 100 °C for 15 min. In this study, the surface sterilization method was modified based on those outlined by Passari et al. (2015) and Purushotham et al. (2018).

The plant tissues, previously disinfected, were cut and diluted in 2 mL of sterile distilled water. At the end of the maceration process, a solution was obtained, which in this case is considered the stock solution of the samples that will undergo subsequent serial dilutions up to 10^−1^ using the suspension-dilution method (Qin et al., 2009). Two different media, supplemented with an antifungal agent, potassium dichromate (K_2_Cr_2_O_7_) at a concentration of 25 μg/mL, were used, as it effectively inhibits fungal growth and restricts the growth of non-actinobacteria (Lu et al., 1959).

The plant tissues were ground separately, and serial dilutions ranging from 10^−1^ to 10^−5^ were prepared in physiological saline solution (NaCl 9 g/L). After vortexing for 5 to10 minutes, 0.1 mL of each dilution was spread onto culture media in Petri dishes. For each dilution and each culture medium, two Petri dishes were seeded, then incubated for 14 days at 28 °C, with inspections conducted every 3 days.

### Screening of endophytic actinobacterial isolates for plant growth promotion traits (PGP)

#### Screening of endophytic actinobacteria able to use rock phosphate (RP) and tricalcium phosphate (TCP) as sole phosphorus source

Selection of actinomycetes able to use RP originating from Khouribga phosphate mine in Morocco (Hamdali et al., 2008b). On the Synthetic Minimum Medium (SMM) containing [10 g/L glucose, 2 g/L NaNO_3_, 0.5 g/L MgSO_4_·7H_2_O, 0.5 g/L KCl, 0.01 g/L FeSO_4_·7H_2_O and K_2_HPO_4_ (0.5 g/L, 4.38 mM)] as described previously containing 0.5 g/L of RP (approximately equivalent to 2.2 mM phosphorus) as a unique P source or on the SMM containing soluble K_2_HPO_4_ (0.5 g/L, 4.38 mM) or no P source. Spores of endophytic actinobacteria isolates showing the most active growth on SMM containing RP as sole P source were subsequently tested for their ability to grow on SMM containing TCP (0.5 g/L, Ca_3_(PO_4_)_2_) (Sigma Aldrich, Isère, France) as the sole P source. Phosphate solubilizing activity was demonstrated on solid Synthetic Minimum Medium (SMM) which contains 0.5% of tricalcium phosphate Ca_3_(PO^4^)_2_ as the sole source of phosphorus. Isolated strains were seeded on Synthetic Minimum Medium (SMM) in tight streaks and are incubated in 7-days (Hamdali et al., 2008b).

#### Quantitative estimation of the amount soluble phosphate released in the growth medium by the selected endophytic actinobacteria isolates

The selected endophytic actinobacteria isolates were inoculated at 10^6^ spores/mL in 250 mL Erlenmeyer flasks containing 50 mL of liquid Synthetic Minimum Medium (SMM) with 0.5 g/L RP or 0.5 g/L TCP as sole P source, in triplicate, and grown for 4 and 7 days at 28 °C on a rotary shaker (180 g/min) (Hamdali et al., 2008b). For TCP and RP solubilization evaluations, 80 mL of each broth was transferred into different 100 mL conical flasks. Since triplicates were carried out for each microbial isolation, there were 150 conical flasks containing 80 mL of culture broth.

The pH value of the SMM culture broths containing tricalcium phosphate and rock phosphate were measured, respectively. Phosphate solubilizing endophytic actinobacteria’s quantitative solubilization capacity of TCP and RP. After 4 and 7 days of incubation at 28 °C under shaking to 150 rpm, 20 mL of actinobacterial suspensions were taken and centrifuged (12,000*g* for 15 min).

Supernatants were recovered and analyzed for their assimilable P content and pH measurements. Assimilable P content was carried out by the ascorbic acid colorimetric method (Murphy and Riley, 1962). About 1 mL of supernatant is mixed with 160 μL of a reaction solution and the OD (Optical Density) was then measured at 880 nm after a few minutes of incubation at room temperature. Phosphate soluble content was calculated based on a standard P solution of KH_2_PO_4_.

#### Estimation of production of phytohormone Indole-3-Acetic Acid

The bacterial suspension of endophytic actinobacteria isolated from the seven isolated strains were inoculated in 10 mL of LB medium, containing 1 mL of 0.1% L-tryptophan and incubated at 30 °C for 7 days at 10000 rpm/10 min at 4 °C and 2 mL of the collected supernatant was incubated in the dark after addition of 4 mL of Salkowski reagent [1 mL of 0.5 M FeCl₃ and 50 mL of 35% perchloric acid (HClO₄)] (Gordon and Weber, 1951; Gang et al., 2019), for 15 min. The IAA production was determined by mixing 1 mL of supernatant and 2 mL of Salkowski reagent [2% FeCl₃·6H₂O and 37% perchloric acid (HClO₄)] and incubated in the dark for 30 min for the development of reddish pink colored complex.

The presence of IAA was visually observed by the reddish pink color and quantified by measuring the absorbance at 530 nm in a UV-Spectrophotometer (Singh and Prasad, 2014). Each experiment was performed in duplicate. The quantity in the culture was determined and expressed as μg/mL.

#### Screening of endophytic actinobacterial isolates from *Anacyclus pyrethrum* for antagonistic activity: antifungal and antibacterial activity of endophytic actinobacteria

The capacity of endophytic actinobacterial strains to inhibit the growth of phytopathogenic fungi will be tested by the direct confrontation method in petri dish on PDA medium (Zhao et al., 2014). This technique consists in streaking the isolates of endophytic actinobacteria on the surface of Bennett agar medium. The plates are then incubated at 28 °C for 21 days. After incubation, 6 mm. agar cylinders are removed with a sterile punch. A 08 mm diameter mycelial pellet taken from the edge of 7-day *Fusarium fujikuroi (Ch3)* fungal colony and then deposited approximately 3 cm from the antagonistic strain being tested. The incubation of the plates is done at 28 °C in the dark for 10 days.

The demonstration of the antibacterial activity of the isolates of endophytic actinobacteria of *Anacyclus pyrethrum* will also be carried out by the agar cylinder method on Bennett medium. This technique consists in streaking the endophytic actinobacteria isolates on the surface of ISP2 agar medium. The plates are then incubated at 28 °C for 21 days (Zhao et al., 2014). After incubation, 6 mm diameter agar cylinders are removed with a sterile punch and placed on the surface of the medium previously inoculated with the test bacteria.

The inoculated plates are left on the bench at room temperature for about 2 h to allow diffusion of the active molecules. They are then incubated at 37 °C for 24 h. Zones of inhibition appear between the edges of the agar cylinders and the limits of the zones where the test bacteria are.

#### Molecular identification of isolated endophytic actinobacteria: 16S rRNA gene sequencing and phylogenetic analysis

The 16S rDNA was amplified with the PCR method using universal primers 27F (AGAGTTTGAMCCTGGCTCAG) and 1492R (GGTTACCTTGTTACGACTT) (Lane, 1991; Delbari et al., 2023).

Amplification was carried out in 25 mL of reaction mixture containing 10 L of AccuPower Taq PCR PreMix (Bioneer, Oakland, CA, USA), 1.25 mol of each primer and 50 ng of DNA.

PCR condition was as follows: after initial denaturation (96 °C for 1 min), 30 cycles of 96 °C for 30 s, 60 °C for 30 s and 72 °C for 1 min 30 s were performed, followed by a final extension (5 min, 72 °C).

Amplification was carried out using a GeneAmp PCR 9700 System (Applied Biosystems, Foster City, CA, USA). Negative controls were included with no addition of template DNA. The amplified products were visualized on a 2% (w/v) agarose gel stained with ethidium bromide. PCR products from each isolate were sequenced using 27F and 1492R primers. Sequence similarity searches were performed against corresponding sequences of members of the Streptomycetaceae family using the online sequence analysis resources LEBIBI database and GenBank sequence database (all nucleotide data should then be deposited in the public domain). The online programme BLASTn is then used to find related sequences with known taxonomic information in the databank at the NCBI website.^1^

Unrooted phylogenetic tree was inferred using the Neighbor-Joining method (Saitou and Nei, 1987). The percentage of replicate tree in which the associated taxa clustered together in the bootstrap test (1,000 replicates) is shown next to the branches (Hatami et al., 2025). The evolutionary distances were computed using the Kimura 2-parameter method (Delbari et al., 2023; Mahdi et al., 2022) and are expressed in number of base substitutions per site. This analysis involved 52 nucleotide sequences. Evolutionary analyses were conducted in MEGA X (Lane, 1991).

## Results

### Isolation of endophytic actinobacteria

**Figure 2:**
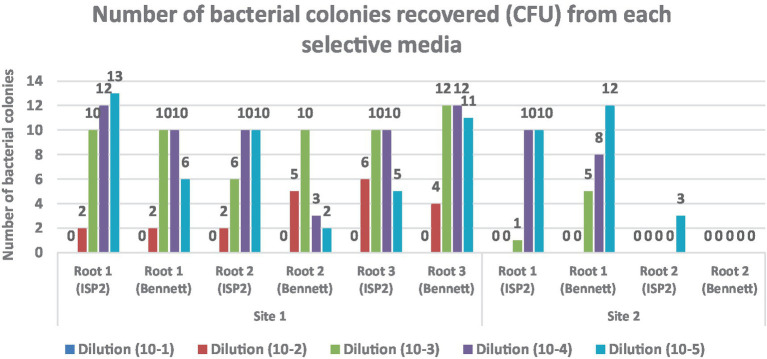
Colony-forming units (CFU/cm^2^) of endophytic actinobacteria isolated from *Anacyclus pyrethrum* roots. The counts were determined using selective media (ISP 2 and Bennett agar).

All presumptive endophytic actinobacterial isolates were subcultured on selective media plates which they have been grown on [International Streptomyces Project medium (ISP 2), Bennett] and the isolates were initially grouped based on morphological characteristics of the colonies (Figure 2).

The surface sterilization protocol was effective, as evidenced by the absence of microbial growth on ISP2 plates after incubation, confirming that the isolated actinobacteria are truly endophytic. Roots, being the most favorable plant organ for microbial colonization, provide a nutrient-rich and protective environment that supports the establishment and proliferation of endophytic actinobacteria (Berendsen et al., 2012; Singh et al., 2021; Gao et al., 2022).

A comparison between samples from two sites 1 and 2 revealed a significant difference in endophytic bacterial abundance. Root samples from Site 1 showed substantial bacterial growth on both ISP2 and Bennett media, with the highest CFU counts at intermediate dilutions.

In contrast, samples from Site 2 exhibited little to no bacterial growth, suggesting a low density or absence of culturable endophytes. Bennett medium supported more abundant bacterial growth than ISP2, particularly in Site 1 samples.

Bacterial growth analysis on ISP2 and Bennett media showed significant differences between Site 1 and Site 2. Root samples from Site 1 exhibited abundant growth across all dilutions, particularly at 10^−2^ and 10^−3^, while Site 2 samples showed little to no growth, indicating a low presence of culturable endophytes. Bennett medium supported more colonies than ISP2, especially for Site 1 samples.

**Figure 3:**
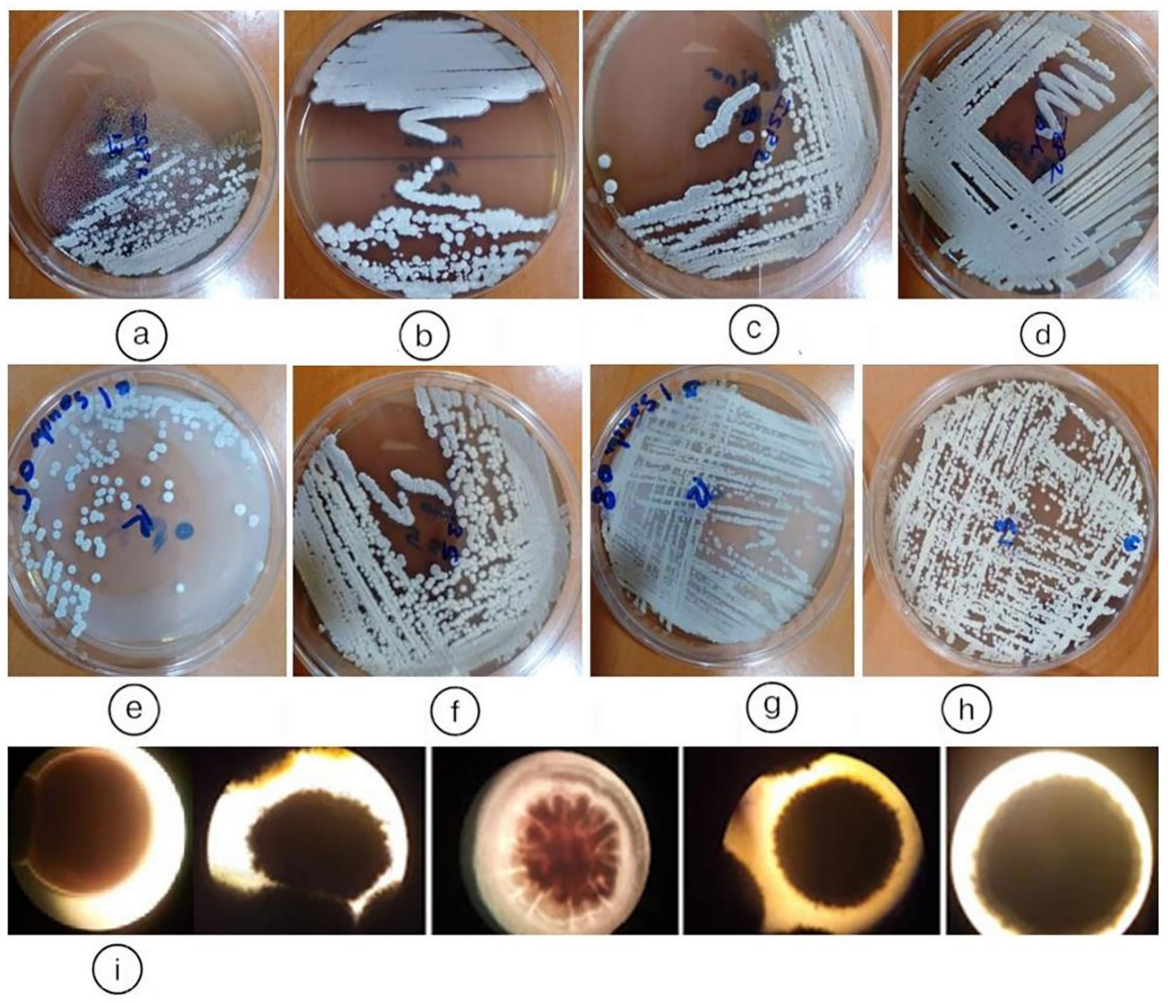
Representative colony morphologies of endophytic actinobacteria isolates from *Anacyclus pyrethrum* roots, grown on ISP2 medium at 28 °C for 7 days: **(a)** *AGS13*; **(b)** *AGS3*; **(c)** *AGS9*; **(d)** *AGS 1*; **(e)**, **(f)** *AGS5 (By5a)*; **(g)** *AGS8 (By8)*; **(h)** *AGS10 (By10)*; **(i)** macroscopic appearance of colonies of endophytic actinobacteria.

Figure 3 illustrates representative colony morphologies of endophytic actinobacteria isolated from *Anacyclus pyrethrum* roots after 7 days of growth on ISP2 medium at 28 °C. Out of 100 isolates, thirteen were preliminarily identified as putative actinobacteria, all derived from root tissues. These isolates were classified into five distinct morphotypes based on colony structure, aerial and substrate mycelium development, and pigment production. Initial characterization relied on macroscopic and microscopic traits such as spore coloration, mycelial organization, and production of diffusible pigments, providing a preliminary classification prior to molecular identification.

### Screening of endophytic actinobacterial isolates for plant growth promotion (PGP) traits

#### Screening of endophytic actinobacteria able to use rock phosphate (RP) and tricalcium phosphate (TCP) as sole phosphorus source

**Figure 4:**
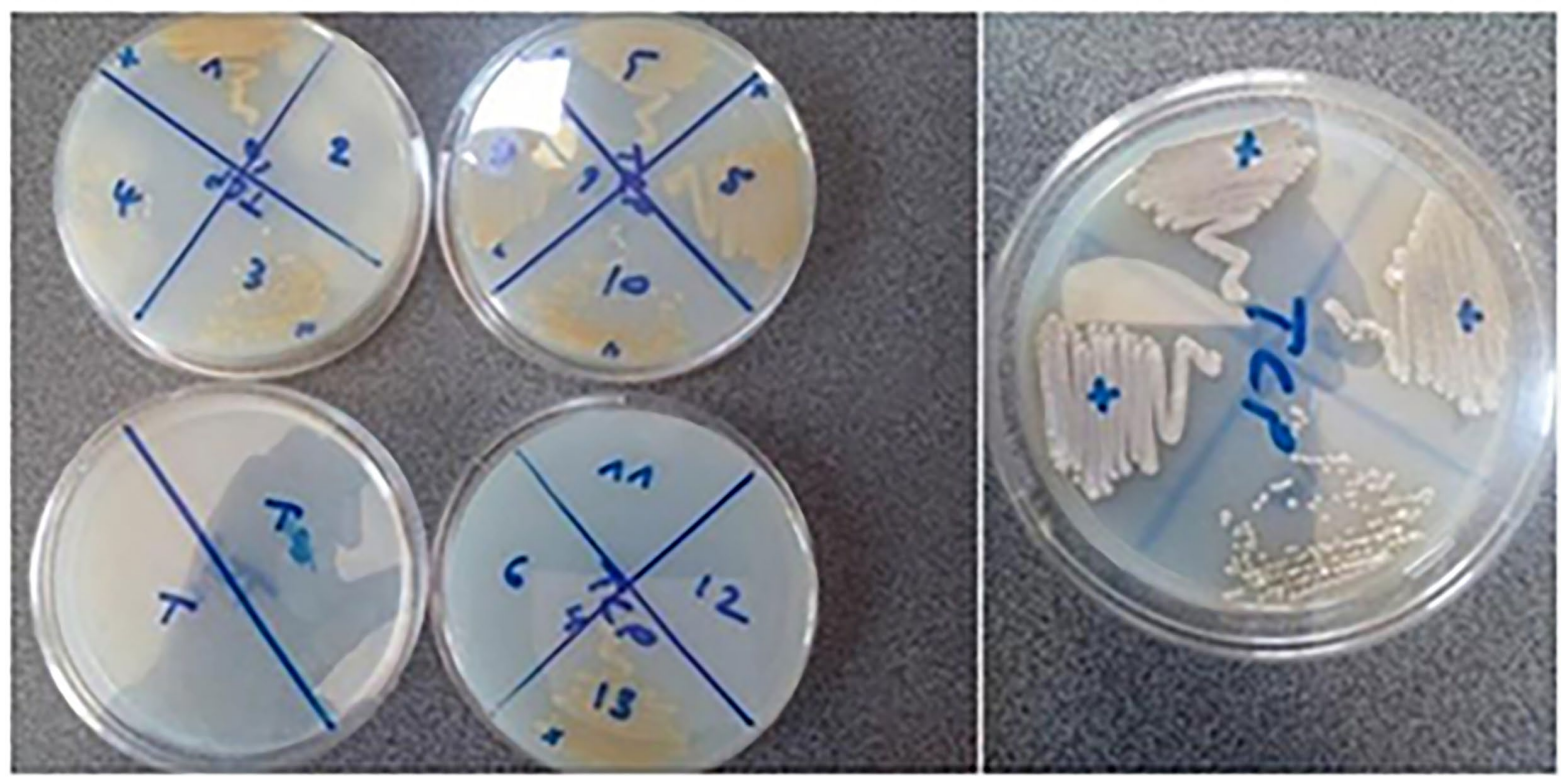
Tri-Calcium Phosphate (TCP) Solubilization by Endophytic Actinobacteria. The figure illustrates the phosphate-solubilizing ability of selected endophytic actinobacterial isolates (*AGS1, AGS3, AGS4, AGS5, AGS8, AGS9, AGS10*, and *AGS13*) grown on solid Synthetic Minimum Medium (SMM) supplemented with 0.5 g/L TCP. After incubation in 7 days, the strains seeded in tight streaks in solid SMM containing TCP are developed.

**Figure 5:**
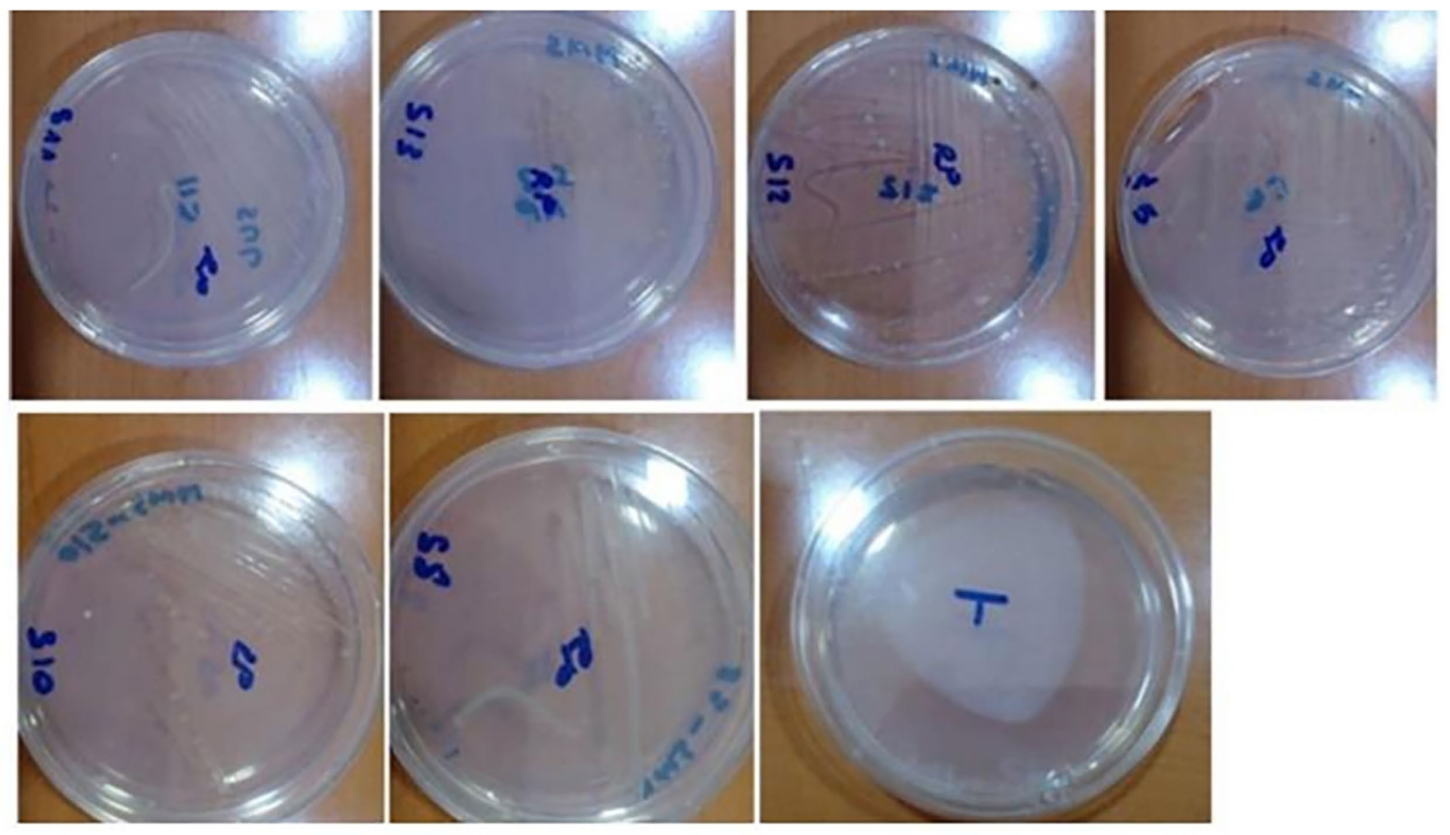
Solubilization of natural Rock Phosphate (RPn) by endophytic actinobacteria. The figure shows the ability of the endophytic isolates (*AGS5, AGS8, AGS10, AGS11, AGS12* and *AGS13*) to solubilize phosphate from natural rock when grown on solid Synthetic Minimum Medium (SMM) supplemented with 0.5 g/L RP. After incubation in 7 days, the strains seeded in tight streaks in solid SMM containing Phosphate rock (RP) are developed.

In order to evaluate their phosphate-solubilizing potential, the selected isolates were tested for their ability to solubilize Tri-Calcium Phosphate (TCP) and natural Rock Phosphate (RPn) on Synthetic Minimum Medium (SMM) (see Figures 4, 5):

#### Quantitative estimation of soluble phosphate released by selected endophytic actinobacterial isolates after 4 and 7 days of growth in liquid Synthetic Minimum Medium (SMM) containing 0.5 g/L TCP and RP

**Figure 6:**
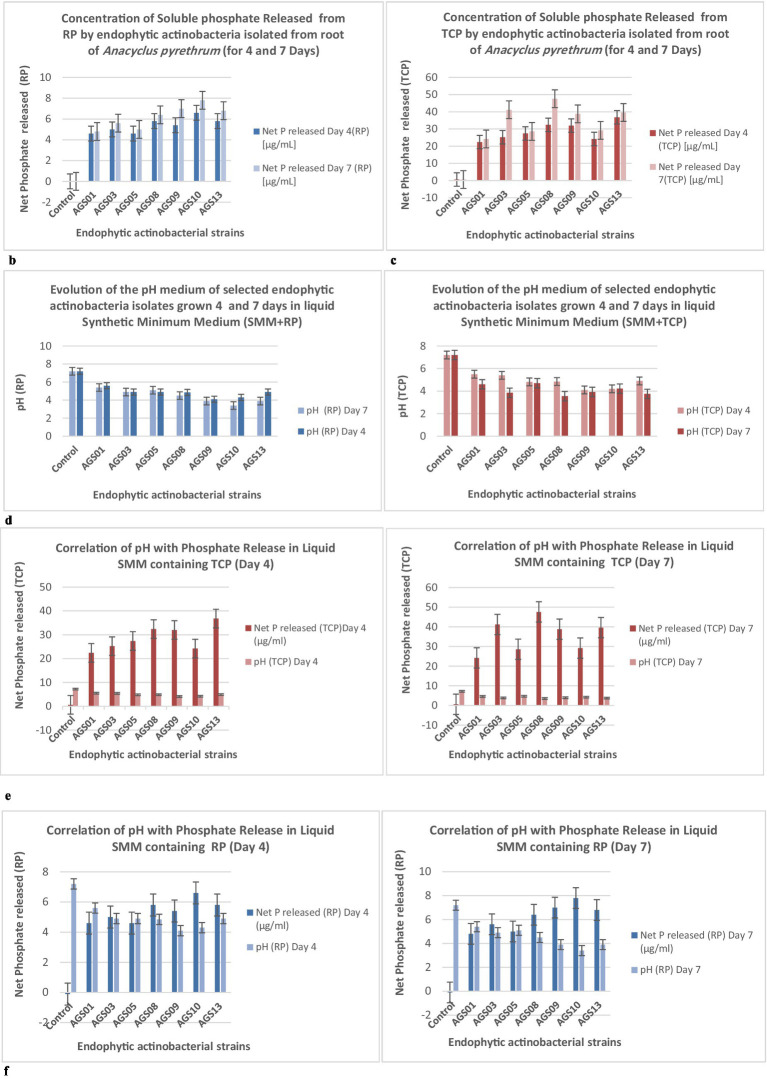
**(b)** Soluble phosphate released (μg/mL) from Tri-Calcium Phosphate (TCP) by endophytic actinobacteria isolates in SMM (0.5 g/L TCP) after 4 and 7 days. Controls are non-inoculated flasks. **(c)** Soluble phosphate released (μg/mL) from natural Rock Phosphate (RP) by endophytic actinobacteria isolates in SMM (0.5 g/L RP) after 4 and 7 days. Controls are non-inoculated flasks. **(d)** pH variation of SMM supplemented with Rock Phosphate (RP) or Tri-Calcium Phosphate (TCP) after 4 and 7 days of growth of actinobacteria isolates. **(e)** Correlation between phosphate (P) release (μg/mL) from Tricalcium Phosphate (TCP) and medium pH in non-inoculated controls and cultures of selected endophytic actinobacteria isolates grown for 4 and 7 days in liquid Synthetic Minimum Medium (SMM) containing 0.5 g/L TCP. **(f)** Correlation between phosphate (P) release (μg/mL) from Natural Rock Phosphate (RP) and medium pH in non-inoculated controls and cultures of selected endophytic actinobacteria isolates grown for 4 and 7 days in liquid Synthetic Minimum Medium (SMM) containing 0.5 g/L RP. Error bars denote standard deviations calculated from two independent culture replicates.

The Figure 6b shows the concentration of soluble phosphate (P) released from tricalcium phosphate (TCP) used as a phosphorus source, measured after 4 and 7 days of incubation in liquid SMM. The results compare non-inoculated flasks (control) with cultures containing endophytic actinomycete isolates: the non-inoculated control shows a very low concentration of soluble P released (0.6 μg/mL), indicating negligible TCP degradation without biological intervention. After 4 days, the concentration of soluble phosphate released ranged from 22.4 μg/mL (*AGS01*) to 36.8 μg/mL (*AGS13*), indicating that some strains (e.g., *AGS13*) have higher TCP solubilization activity. After 7 days, the release of soluble P increased significantly, reaching maximum values of 47.6 μg/mL (*AGS08*), followed by 39.6 μg/mL (*AGS13*). The isolates *AGS08* and *AGS13* stand out for their efficiency in releasing soluble phosphate.

The Figure 6c shows the concentration of soluble phosphate (P) released from natural rock phosphate (RP) used as a phosphorus source, measured after 4 and 7 days of incubation in liquid SMM. The results compare non-inoculated flasks (control) with cultures containing selected endophytic actinomycete isolates (Figure 6c): the non-inoculated control shows a value of −0.104 μg/mL which may be attributed to minor measurement errors or minimal chemical activity without inoculation. After 4 days, the released P concentrations ranged from 4.6 μg/mL (*AGS01* & *AGS03*) to 6.6 μg/mL (*AGS10*), with moderate differences between isolates. After 7 days, phosphate concentrations slightly increased, reaching a maximum of 7.8 μg/mL (*AGS10*), followed by 6.8 μg/mL (*AGS13*). Isolates *AGS10* and *AGS13* stood out for their higher capacity to solubilize rock phosphate. The solubilization of rock phosphate (RP) is lower than that of TCP, which is expected, as TCP is more easily solubilized due to its chemical structure. Although rock phosphate solubilization by the isolates is lower than that of TCP, certain isolates, particularly *AGS10* and *AGS13*, show promising potential for improving phosphorus availability from less soluble natural sources.

The pH (TCP) showed a general trend of decreasing as the strains grew, which is an indicator of bacterial metabolic activity and organic acid production and decreases for all strains between Day 4 and Day 7, with the lowest pH values reached by strains *AGS08* (3.55) and *AGS03* (3.85) at the end of the incubation. A clear correlation can be observed between the pH drop and the increase in phosphate release. Strains that lowered the pH the most also released the highest amounts of phosphate. For Natural Rock Phosphate (RP), the pH drop is also present for RP but is less pronounced compared to TCP. For example, *AGS10* and *AGS09* have relatively low pH values (3.9 and 3.4, respectively) after 7 days, but phosphate release remains relatively low. Although the pH decreases, phosphate solubilization is less effective. In general, pH values are consistently lower at Day 7 compared to Day 4 (Figure 6d).

The Figure 6e shows the correlation between the pH of the medium and the amount of phosphorus (P) released from tricalcium phosphate (TCP) by endophytic actinobacteria isolates (*AGS01* to *AGS13*): in the control sample, no significant P release was observed (0.6 μg/mL), and the pH remained constant at 7.2. The isolates showed varying capacities to release phosphorus, influencing the pH. Higher phosphate release corresponds to a decrease in pH (e.g., *AGS13* with 36.8 μg/mL released P has a pH of 4.9) (Day 4). The trend remains similar, with isolates showing higher P release (e.g., *AGS13* with 39.6 μg/mL) correlating with lower pH (3.75) (Day 7). *AGS13* stands out as the most efficient in both P release and pH reduction. Other effective isolates include *AGS09* & *AGS10*.

The Figure 6f displays the correlation between pH and the amount of phosphorus (P) released from natural rock phosphate (RP) by endophytic actinobacteria isolates (*AGS01* to *AG13*): the control shows no significant P release 0 μg/mL and maintains a pH of 7.2. The isolates show varying capabilities to solubilize RP, which corresponds to changes in pH. A clear trend shows that higher P release correlates with lower pH values (Day 4). For example, AG13 shows the highest P release (6.6 μg/mL) and a reduced pH of 4.9. The trend is consistent, with isolates releasing more P (e.g., *AG13* with 7.8 μg/mL) showing lower pH values (3.9) (Day 7).

The correlation between phosphate release and pH drop is particularly evident in liquid Synthetic Minimum Medium (SMM) containing TCP. Strains that released more phosphate (such as *AGS08* & *AGS03*) also caused greater acidification of the SMM. This acidification is less pronounced in RP but still observable, indicating that organic acid production occurs for both phosphate sources, with TCP likely being more soluble.

### Estimation of production of phytohormone Indole-3-Acetic Acid

The Indole Acetic Acid assay is performed after 4 days of incubation by applying the colorimetric method described by Gordon and Weber (1951) and Brick et al. (1991). Salkowski is a coloring reagent that can be used to test for IAA content (Saitou and Nei, 1987). Salkowski in this study consisted of ferric chloride (FeCl_3_) and perchloric acid (HClO₄) (Salkowski, 1929). Interaction between IAA and Fe to form complex compounds [Fe_2_(OH)_2_(IA)_4_], IA is indole-3 acetate. The complex formed will produce a pink color (Salkowski, 1929). The ability of the endophytic actinobacteria to produce Indole Acetic Acid was then determined by visually comparing the pink color intensity of the medium. A medium color change from the pink to pink-red indicated the presence of IAA due to the conversion of L-tryptophan to Indole Acetic Acid by the endophytic actinobacteria (Gordon and Weber, 1951; Salkowski, 1929).

The optical density is measured by spectrophotometer at *λ* = 530 nm. The amounts of IAA produced are calculated from the calibration curve equation from Patel and Patel (2014) (Dilution 1/3, Blanc = 0.254 nm).

**Figure 7:**
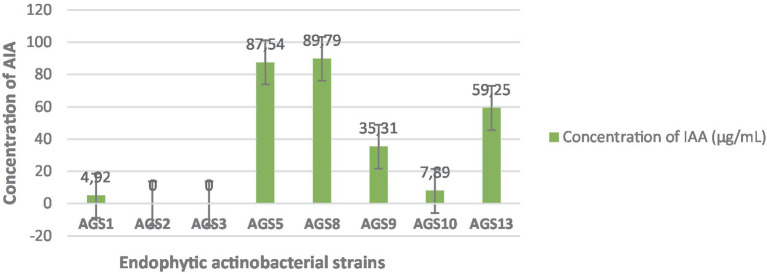
Quantitative estimation of Indole-3-Acetic Acid (IAA) production by the endophytic isolates. Error bars represent the standard deviations of the means from two independent replicates.

The Figure 7 presents the quantitative estimation of Indole-3-Acetic Acid (IAA) production by 08 endophytic actinobacteria isolates. A positive correlation between absorbance and concentration is observed in several of the strains, indicating that as the concentration of the substance increases, the absorbance at 530 nm also rises. This trend is typical in spectrophotometry, where higher concentrations of a substance tend to absorb more light, leading to increased absorbance (Skoog et al., 2013). For instance, strains *AGS5* displays a high absorbance of 0.75 and a corresponding concentration of 87.54 μg/mL, while *AGS8* exhibits an even higher absorbance of 0.786 with a concentration of 89.79 μg/mL. *AGS9* demonstrates a moderate absorbance of 0.327 paired with a concentration of 35.31 μg/mL. Additionally, *AGS10* exhibits an unusually low absorbance of 0.105, despite a concentration of 7.89 μg/mL, which is lower than other strains with comparable concentrations. This could imply that the substance in *AGS2* has different light absorption properties or that a measurement discrepancy occurred. The results highlight *AGS8* and *AGS5* as a promising strain for AIA production, followed by *AGS9*.

### Screening of endophytic actinobacterial isolates from *Anacyclus pyrethrum* for antagonistic activity: antifungal activity of endophytic actinobacteria

**Figure 8:**
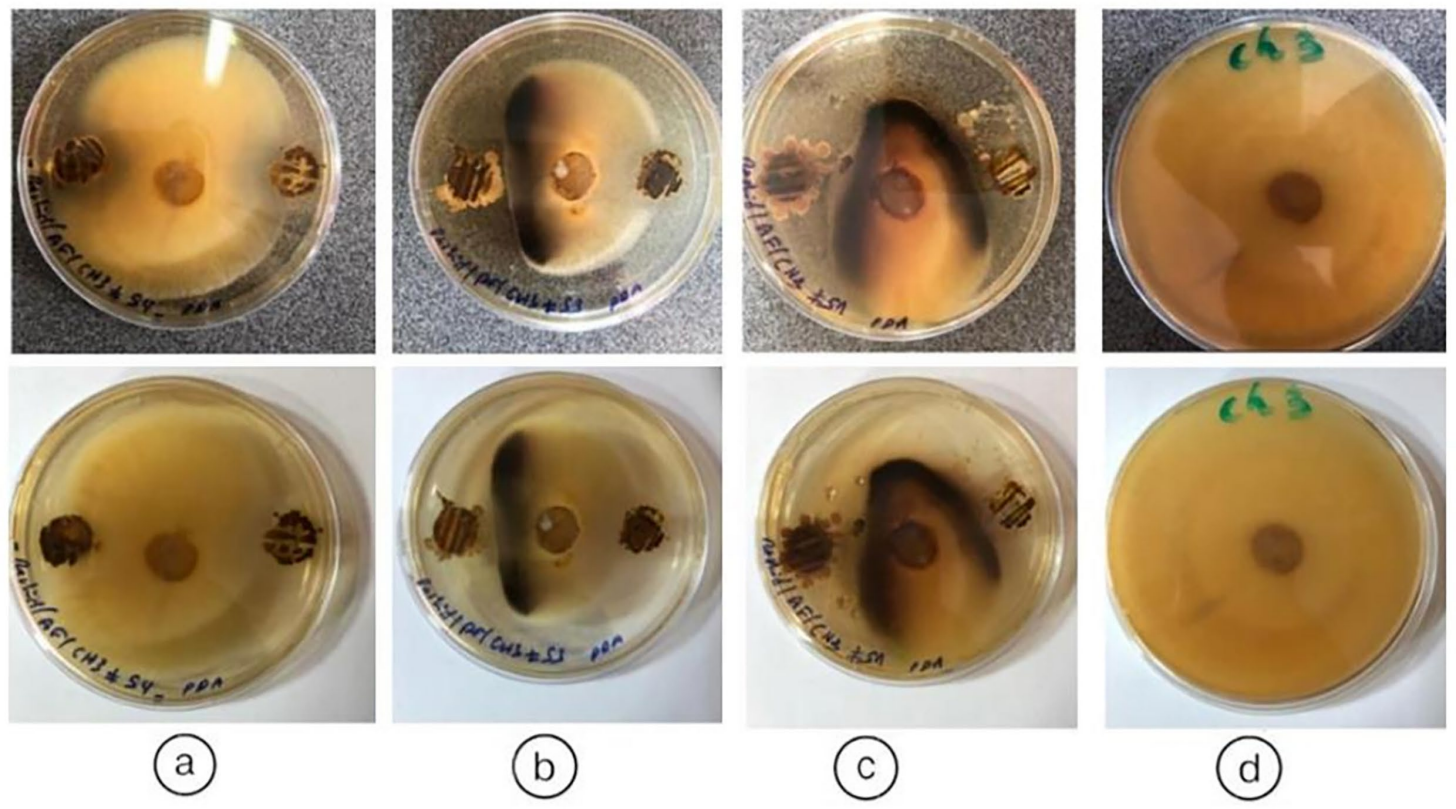
Antifungal activity of endophytic actinobacteria isolates (*AGS5, AGS8*, and *AGS10*) obtained from *Anacyclus pyrethrum*, showing growth inhibition of pathogenic fungi. **(a)** *AGS5 (By5a)*; **(b)** *AGS8 (By8)*; **(c)** *AGS10 (By10)*; **(d)** untreated control.

The interpretation of antifungal activity in Figure 8 is based on the observation of inhibition zones around the actinobacteria colonies. In several Petri dishes, clear zones are observed around the bacterial spots, indicating inhibition of fungal growth. These zones vary in size, suggesting differences in the effectiveness of actinobacteria isolates against the tested fungi. Thirteen (13) isolates were tested for their ability to inhibit the fungal phytopathogen *Fusarium fujikuroi* (Ch3) using a dual culture *in vitro* assay. Among these, three (3) isolates (*AGS5, AGS8, AGS10*) showed antifungal activity against the tested pathogens (Figure 8).

### Antibacterial activity of endophytic actinobacteria

The test revealed significant variability in the results. Diameters ranged from 15 to 18 mm for *E. coli*, from 16 to 25 mm for *K. pneumoniae*, and from 16 to 18 mm for *S. aureus*. Isolates *AGS2, AGS3, AGS4, AGS6, AGS7, AGS11,* and *AGS12* exhibited no inhibitory activity against the test bacteria. Strain *AGS8* showed the strongest inhibitory effect, with a diameter of 25 mm against *K. pneumoniae*. The antagonistic activity of isolated endophytic actinobacteria against clinically relevant bacteria was assessed using the agar cylinder method.

**Figure 9:**
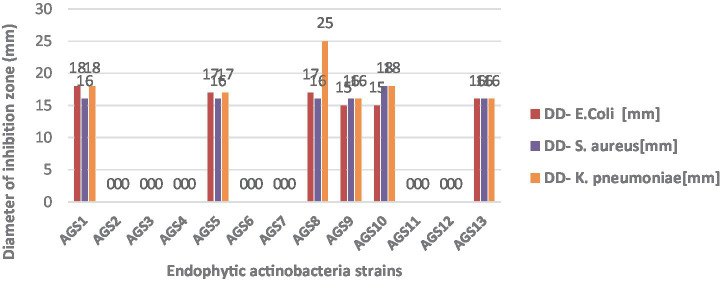
Antibacterial activity of endophytic actinobacteria isolates from *Anacyclus pyrethrum.*

Out of the actinobacterial strains tested, 6 strains (*AGS1, AGS5, AGS8, AGS9, AGS10*, and *AGS13*) demonstrated activity against Gram-positive bacteria (*Staphylococcus aureus*), representing 33.33% of the strains. The remaining strains (*AGS1, AGS5, AGS8, AGS9, AGS10*, and *AGS13*), accounting for 66.66%, showed activity against Gram-negative bacteria (*E. coli* and *Klebsiella pneumoniae*). Both *Staphylococcus aureus*, *Escherichia coli*, and *Klebsiella pneumoniae* were found to be sensitive to *Anacyclus pyrethrum* macerate at a concentration of 100 μg/mL (Figure 9).

### Molecular identification of isolated endophytic actinobacteria: 16S rRNA gene sequencing and phylogenetic analysis

**Figure 10:**
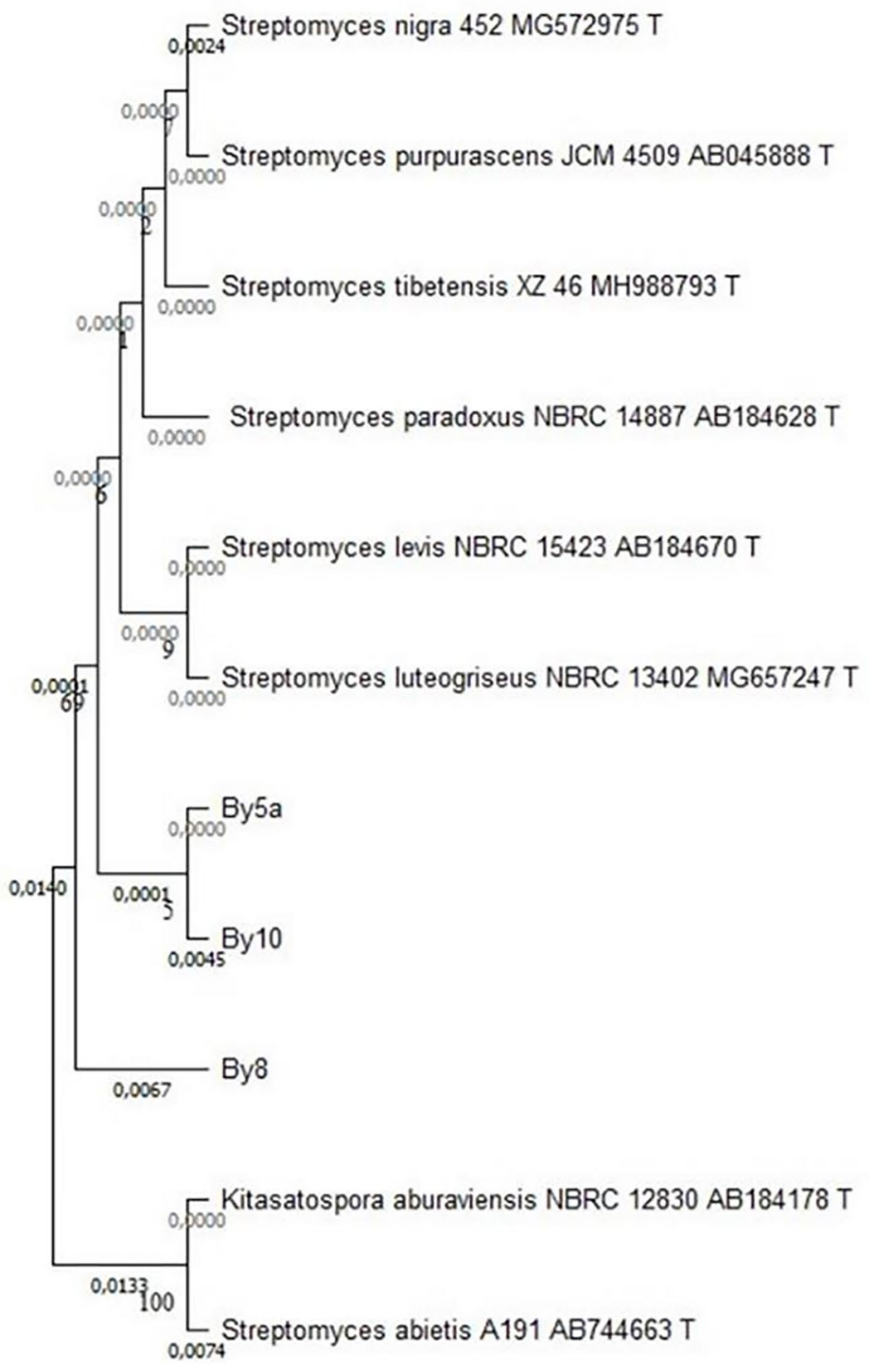
Phylogenetic tree based on 16S rRNA gene sequences, constructed using the Neighbor-Joining method. The tree illustrates the relationships between the *Streptomyces* isolates *By5a*, *By10*, and *By8* and selected reference species from the Streptomycetaceae family. Bootstrap values (expressed as percentages) are shown at the nodes to indicate the reliability of each branch, with values >70% considered strongly supported. The branch lengths are proportional to the genetic distances, highlighting the close relationship between *By5a* and *By10* and the slightly higher divergence of *By8*.

16S rRNA gene analysis revealed that the root-associated *Streptomyces* isolates from *Anacyclus pyrethrum* belong to the Streptomycetaceae family. Phylogenetic and pairwise sequence analyses showed strong similarity, with By5a and By10 clustering closely, while By8 displayed only minor divergence, indicating low genetic variation within this lineage (see Figure 10).

Sequence analysis revealed that isolates *By5a* and *By10* were nearly identical, exhibiting an extremely low genetic distance (0.0001) and an estimated sequence similarity of approximately 99.99%. In contrast, isolate *By8* showed a slightly lower similarity to *By10* (99.55%) with a corresponding genetic distance of 0.0045, suggesting a modest level of intra-group variation. Phylogenetic comparisons with type strains indicated that the three isolates (*By5a, By10,* and *By8*) were most closely related to *Streptomyces luteogriseus* (99.33% similarity, genetic distance of 0.0067), while more distantly related to *S. levis* (99.01%, distance 0.0099) and *S. paradoxus* (99.00%, distance 0.0100) (Table 1).

**Table 1:** Molecular characterization and genetic relationships of root-endophytic actinobacterial *Streptomyces* isolates from *Anacyclus pyrethrum*.

Isolate and comparison	Closest type strain (NCBI-GenBank accession)	Accession number	Genetic distance	% Similarity
*AGS5 (By5a)*	*Streptomyces variabilis* NBRC 12825	AB184884	–	–
*AGS10 (By10)*	*Streptomyces variabilis* NBRC 12825	AB184884	–	–
*AGS8 (By8)*	*Streptomyces albogriseolus* NRRL B-1305	AJ494865	–	–
*By5a & By10*	–	–	0.0001	99.99%
*By10 & By8*	–	–	0.0045	99.55%
*(By5a, By10, By8) & S. luteogriseus*	–	–	0.0067	99.33%
*(By5a, By10, By8) & S. levis*	–	–	0.0099	99.01%
*(By5a, By10, By8) & S. paradoxus*	–	–	0.0100	99.00%

## Discussion

The surface sterilization procedure proved successful, as control plates showed no microbial growth, indicating that the microorganisms recovered originated from within the plant tissues rather than from external contaminants. This confirms their identity as true endophytes, aligning with recent studies that highlight the importance of stringent sterilization for accurate isolation of endophytic bacteria (Singh et al., 2021; Gao et al., 2022). The root tissues were identified as the main colonization site, probably because their nutrient-rich exudates create a stable internal environment that protects endophytes from external stresses and microbial competition, thereby promoting their establishment (Li et al., 2023).

Significant differences in microbial abundance were observed between sampling sites 1 & 2. Roots from Site 1 yielded higher colony-forming units (CFUs) on ISP2 and Bennett media, particularly at intermediate dilutions (10^−2^ and 10^−3^), whereas Site 2 samples produced minimal growth. This reveals that environmental factors such as soil composition, climate, and host physiology strongly influence endophytic density and diversity, aligning with previous findings on ecological influences on microbial communities (Olanrewaju and Babalola, 2021). In addition, Bennett medium supported greater recovery than ISP2, highlighting the role of culture medium composition in selectively enriching specific microbial groups (Ait Assou et al., 2025).

From 100 isolates, thirteen were preliminarily identified as putative actinobacteria from roots, grouped into five morphotypes based on colony features such as spore pigmentation, mycelial organization, and diffusible pigments. Morphological characterization, while informative, is limited for taxonomic resolution, reinforcing the need for molecular approaches like 16S rRNA sequencing to confirm identity and phylogeny (Wang et al., 2021). Overall, Site 1 roots contained a richer and more active endophytic actinobacterial community, emphasizing the context-dependence of microbial diversity shaped by host genotype, soil properties, and microclimate (Li et al., 2023).

Several isolates exhibited the ability to solubilize phosphate when streaked on solid SMM containing TCP, showing growth on both TCP and RP media, most likely through mechanisms involving acidification and enzymatic activity (Jiang et al., 2021; Liu et al., 2022; Yuan et al., 2022). Tri-Calcium Phosphate (TCP) solubilization was generally higher than Rock Phosphate (RP), reflecting differences in mineral composition and acidification requirements (Chauhan et al., 2022). Isolates *AGS08* and *AGS13* demonstrated the highest soluble phosphorus release (up to 47.6 μg/mL for TCP), with corresponding pH reductions (*AGS08*: 3.55; *AGS13*: 3.75). This activity is mainly attributed to acidification through the secretion of organic acids, as well as enzymatic processes, confirming recent findings that acidification is a key mechanism in phosphate solubilization (Fankem et al., 2022). These results highlight their potential as biofertilizers in phosphorus-deficient soils (Pang et al., 2024).

Antifungal assays showed that three isolates (*AGS5*, *AGS8*, *AGS10*) inhibited *Fusarium fujikuroi*, with variable inhibition zones indicating strain-specific metabolite production. Observed pathogen stress responses suggest the action of diffusible antifungal compounds or lytic enzymes, confirming the biocontrol potential of these endophytes (Verma et al., 2021; Sharma et al., 2022; Jiao et al., 2022).

Selective antibacterial activity was also recorded: six isolates (*AGS1, AGS5, AGS8, AGS9, AGS10, AGS13*) inhibited both Gram-positive and Gram-negative bacteria, with *AGS8* showing the strongest effect against *K. pneumoniae* (25 mm zone). These results reinforce their role in indirect plant protection via biocontrol, offering eco-friendly alternatives to chemical pesticides (Singh et al., 2022; Xu et al., 2023).

The quantitative assessment of Indole-3-Acetic Acid (IAA) production revealed that *AGS8* and AGS5 produced the highest concentrations (89.79 and 87.54 μg/mL, respectively), supporting their potential as plant growth-promoting biofertilizers. The differences observed among strains highlight the need to carefully select the most effective isolates for applications in sustainable agriculture (Chand et al., 2020).

This study demonstrates that endophytic actinobacteria isolated from *Anacyclus pyrethrum* roots possess diverse functional traits, including phosphate solubilization, antimicrobial activity, and phytohormone production. These characteristics highlight their dual role in enhancing plant growth and providing biocontrol, as well as their potential as sustainable alternatives to chemical fertilizers and pesticides. Recent studies have further confirmed the multifunctional benefits of endophytic actinobacteria in agriculture, such as promoting plant growth, improving soil health, and controlling plant pathogens, suggesting that their integration into cropping systems can offer environmentally friendly and cost-effective strategies for sustainable agriculture (Mohamad et al., 2022).

The 16S rRNA gene analysis of the three root-associated Streptomyces isolates (*AGS5/By5a*, *AGS10/By10*, and *AGS8/By8*) demonstrated a high level of genetic relatedness, with By5a and By10 sharing almost identical sequences (99.99%), while By8 displayed a slightly lower similarity (99.55% with By10). The analysis reveals that the isolates belong to a closely related phylogenetic cluster within the genus *Streptomyces*. Nevertheless, it is well established that 16S rRNA gene sequencing alone does not provide sufficient resolution for reliable species-level identification in *Streptomyces*. Thus, while 16S rRNA analysis remains useful for preliminary classification and phylogenetic inference, its limitations highlight the need for complementary genomic and phenotypic approaches to ensure precise taxonomic assignment. The high sequence similarity among the three isolates reinforces their close evolutionary affiliation, yet comprehensive analyses integrating multilocus sequence data and genomic comparisons are essential for accurately delineating diversity within this complex genus (Ranjan et al., 2023).

The *Streptomyces* isolates characterized in this study exhibited multiple plant growth-promoting (PGP) traits, including phytohormone production and phosphate solubilization and antimicrobial activities. These characteristics are consistent with the beneficial roles reported for endophytic actinobacteria (Gopalakrishnan et al., 2015; Qin et al., 2011). The combination of genetic diversity and functional potential observed among the isolates underscores their promise as sustainable alternatives to synthetic fertilizers and chemical pesticides, offering environmentally friendly strategies for enhancing plant growth and resilience.

## Conclusion

Medicinal plants such as *Anacyclus pyrethrum* serve as reservoirs of diverse endophytic actinobacteria, exhibiting multifunctional traits that can improve plant growth, enhance nutrient availability, and increase resistance to pathogens. Previous studies have reported that such endophytes can produce phytohormones, solubilize phosphate, and exhibit antifungal activity, highlighting their potential as sustainable alternatives to chemical fertilizers and pesticides. The genetic diversity observed among these strains further supports their application as versatile bioinoculants in agriculture.

In the present study, several endophytic actinobacteria were successfully isolated and characterized from the roots of *A. pyrethrum*, confirming their multifunctional potential.

Phylogenetic analyses demonstrated that isolates *By5a, By10*, and *By8* form a monophyletic group closely related to species of the genus *Streptomyces*. *In vitro* assays revealed significant antibacterial and antifungal activities, indicating their potential protective role against a range of plant pathogens.

Overall, these findings highlight the promise of *A. pyrethrum* endophytic actinobacteria as both biofertilizers and biocontrol agents, contributing to sustainable, autonomous, and resilient agricultural systems. Future studies will focus on agronomic trials to assess the impact of these endophytic actinobacteria on maize growth and development, including their interactions with mineral fertilizers and other sustainable agricultural practices. Furthermore, the bioactive metabolites produced by these strains offer promising opportunities for therapeutic and environmental applications.

## Data Availability

The original contributions presented in the study are included in the article/supplementary material, further inquiries can be directed to the corresponding author.

## Footnotes

1
http://www.ncbi.nml.nih.gov/BLAST
