# Correction: Unlocking plant growth-promoting traits of endophytic actinobacteria isolated from *Anacyclus pyrethrum*, an endemic medicinal plant of the Aguelmam azegza region, Morocco

**DOI:** 10.3389/fmicb.2026.1890923

**Published:** 2026-06-18

**Authors:** Rachid Aguennouz, Yassine Aallam, Abdelmajid Haddioui, Hanane Hamdali

**Affiliations:** 1 Laboratory of Agro-Industrial and Medical Biotechnology, Faculty of Sciences and Technology, University of Sultan Moulay Slimane, Beni-Mellal, Morocco; 2 Agro-Biosciences Program, College of Sustainable Agriculture and Environmental Sciences, Mohammed VI Polytechnic University, Ben Guerir, Morocco

**Keywords:** pyrethrum, endophytic actinobacteria, plant growth promoting (PGP), phosphate solubilization, Indole-3-Acetic Acid (IAA), antimicrobial activity

In the published article, four references were incorrect and have been removed. Correct references have replaced the previous incorrect references.

The incorrect reference:

Bhatti, Z. A., Rahman, U., Iqbal, A., and Shah, M. (2022). Effect of culture medium composition on recovery and diversity of actinobacteria from plant roots. *Microbiol. Res*. 257:126998. doi: 10.1016/j.micres.2022.126998.

Is replaced by:

Ait Assou, S., Anissi, J., Dufossé, L., Fouillaud, M., and EL Hassouni, M. (2025). Inducing and enhancing antimicrobial activity of mining-soil-derived actinomycetes through component modification of Bennett's culture medium. *Microbiol. Res*. 16:72. doi: 10.3390/microbiolres16040072.

The new citation has now been inserted in **Discussion**, page 11 and should read:

“In addition, Bennett medium supported greater recovery than ISP2, highlighting the role of culture medium composition in selectively enriching specific microbial groups (Ait Assou et al., 2025)”.

The incorrect reference:

Abo-Zaid, G. A., Mohamed, H. A., and Abdelhameid, N. A. (2022). Endophytic actinobacteria as plant growth-promoting and biocontrol agents. *J. Appl. Microbiol*. 132, 3925–3936. doi: 10.1111/jam.15494.

Is replaced by:

Passari, A. K., Mishra, V. K., Singh, G, Singh, P., Kumar, B., Gupta, V. K., et al. (2017). Insights into the functionality of endophytic actinobacteria with a focus on their biosynthetic potential and secondary metabolites production. *Sci. Rep*. 7:11809. doi: 10.1038/s41598-017-12235-4.

The new citation has now been inserted in **Introduction**, page 2 and should read:

“Endophytic actinobacteria can promote plant growth directly, through the production of phytohormones and solubilization of minerals like phosphorus (Singh et al., 2023), or indirectly, by protecting plants against phytopathogens via antimicrobial production and induction of host resistance (Passari et al., 2017).”

The incorrect reference:

Anavadiya B., Patel A., and Patel A. (2024). Exploring endophytic actinomycetes: a rich reservoir of diverse antimicrobial compounds for combatting global antimicrobial resistance. Microb. Pathog. 177:105015. doi: 10.1016/j.micpath.2023.105015.

Is replaced by:

Mohamad, O. A. A., Liu, Y.-H., Li, L., Ma, J.-B., Huang, Y., Gao, L., et al. (2022). Synergistic plant-microbe interactions between endophytic actinobacteria and their role in plant growth promotion and biological control of cotton under salt stress. *Microorganisms* 10:867. doi: 10.3390/microorganisms10050867.

The new citation has now been inserted in **Discussion**, page 12 and should read:

“…such as promoting plant growth, improving soil health, and controlling plant pathogens, suggesting that their integration into cropping systems can offer environmentally friendly and cost-effective strategies for sustainable agriculture (Mohamad et al., 2022)”.

The incorrect reference:

Boubekri K., Sahli F., Mansouri S., Benali S. (2023). Endophytic actinobacteria as biofertilizers in phosphorus-deficient soils. Frontiers in Sustainable Food Systems 7:1078993. doi: 10.3389/fsufs.2023.1078993.

Is replaced by:

Pang, F., Li, Q., Solanki, M. K., Wang, Z., Xing, Y. X., and Dong, D. F. (2024). Soil phosphorus transformation and plant uptake driven by phosphate-solubilizing microorganisms. *Front. Microbiol*. 15:1383813. doi: 10.3389/fmicb.2024.1383813.

The new citation has now been inserted in **Discussion**, page 12 and should read:

“These results highlight their potential as biofertilizers in phosphorus-deficient soils (Pang et al., 2023).”

The original version of this article has been updated.

